# Supplementary figures and images for: 3D skeletal muscle fascicle engineering is improved with TGF-β1 treatment of myogenic cells and their co-culture with myofibroblasts
Source: PeerJ. 2018 Jul 11;6:e4939. doi: 10.7717/peerj.4939 (PMC6045923; doi:10.7717/peerj.4939)

$\alpha$ -SMA

Merged

FibCon

A

B

C

D

FibTGF $\beta$

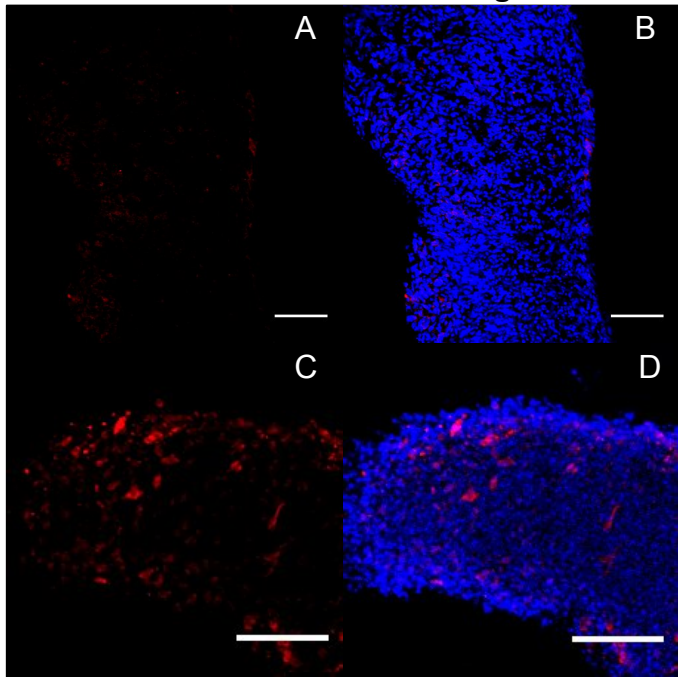

Supplement: Figure S1 — 3D FibCon (A, B) and FibTGFβ (C, D) tissues with α-SMA staining (A, C) and α-SMA merged with DAPI (B, D). Scale bars = 100 μm, 30 μm thick sections. [file peerj-06-4939-s011.pdf]
