# Supplementary material for: 3D skeletal muscle fascicle engineering is improved with TGF-β1 treatment of myogenic cells and their co-culture with myofibroblasts
Source: PeerJ. 2018 Jul 11;6:e4939. doi: 10.7717/peerj.4939 (PMC6045923; doi:10.7717/peerj.4939)
Supplement: Table S1 — Human and mouse GAPDH were used as reference genes for species-specific gene expression. [file peerj-06-4939-s012.docx]

| *Species* | *Gene* | *Forward primer* | *Reverse primer* |
| --- | --- | --- | --- |
| Human | | | |
|  | GAPDH | CCCCCGGTTTCTATAAATTGAGC | AAGAAGATGCGGCTGACTGT |
|  | α-SMA | CTTCCCTGAACACCACCCAGT | CCTCTTCTTCACACATAGCTGGA |
|  | Collagen I A I | TCCCCAGCCACAAAGAGTCTA | CATGGTACCTGAGGCCGTTC |
| Mouse | | | |
|  | GAPDH | CCAGCAAGGACACTGAGCAA | CCCTAGGCCCCTCCTGTTAT |
|  | Myogenin | GCCATCCAGTACATTGAGCG | TGGACGTAAGGGAGTGCAGA |
